# Changes in epicardial and visceral adipose tissue depots following bariatric surgery and their effect on cardiac geometry

**DOI:** 10.3389/fendo.2023.1092777

**Published:** 2023-01-25

**Authors:** J. A. Henry, I. Abdesselam, O. Deal, A. J. Lewis, J. Rayner, M. Bernard, A. Dutour, B. Gaborit, F. Kober, A. Soghomonian, B. Sgromo, J. Byrne, T. Bege, S. Neubauer, B. A. Borlaug, O. J. Rider

**Affiliations:** ^1^ Oxford Centre for Clinical Magnetic Resonance Research, Division of Cardiovascular Medicine, Radcliffe Department of Medicine, University of Oxford, Oxford, United Kingdom; ^2^ Aix-Marseille Univ, CNRS, CRMBM, Marseille, France; ^3^ Aix-Marseille Univ, APHM, INSERM, INRAE, C2VN, Department of Endocrinology, Metabolic Diseases and Nutrition, Marseille, France; ^4^ Department of Upper GI Surgery, Churchill Hospital, Oxford, United Kingdom; ^5^ Division of Surgery, University Hospital Southampton NHS Foundation Trust, Southampton, United Kingdom; ^6^ Aix-Marseille Univ, APHM, Department of Digestive Surgery, Hôpital Nord, Marseille, France; ^7^ Department of Cardiovascular Medicine, Mayo Clinic, Rochester, MN, United States

**Keywords:** obesity, epicardiac adipose tissue, weight loss, bariatric surgery, cardiac remodelling, cardiac geometry

## Abstract

**Introduction:**

Obesity affects cardiac geometry, causing both eccentric (due to increased cardiac output) and concentric (due to insulin resistance) remodelling. Following bariatric surgery, reversal of both processes should occur. Furthermore, epicardial adipose tissue loss following bariatric surgery may reduce pericardial restraint, allowing further chamber expansion. We investigated these changes in a serial imaging study of adipose depots and cardiac geometry following bariatric surgery.

**Methods:**

62 patients underwent cardiac magnetic resonance (CMR) before and after bariatric surgery, including 36 with short-term (median 212 days), 37 medium-term (median 428 days) and 32 long-term (median 1030 days) follow-up. CMR was used to assess cardiac geometry (left atrial volume (LAV) and left ventricular end-diastolic volume (LVEDV)), LV mass (LVM) and LV eccentricity index (LVei – a marker of pericardial restraint). Abdominal visceral (VAT) and epicardial (EAT) adipose tissue were also measured.

**Results:**

Patients on average had lost 21kg (38.9% excess weight loss, EWL) at 212 days and 36kg (64.7% EWL) at 1030 days following bariatric surgery. Most VAT and EAT loss (43% and 14%, p<0.0001) occurred within the first 212 days, with non-significant reductions thereafter. In the short-term LVM (7.4%), LVEDV (8.6%) and LAV (13%) all decreased (all p<0.0001), with change in cardiac output correlated with LVEDV (r=0.35,p=0.03) and LAV change (r=0.37,p=0.03). Whereas LVM continued to decrease with time (12% decrease relative to baseline at 1030 days, p<0.0001), both LAV and LVEDV had returned to baseline by 1030 days. LV mass:volume ratio (a marker of concentric hypertrophy) reached its nadir at the longest timepoint (p<0.001). At baseline, LVei correlated with baseline EAT (r=0.37,p=0.0040), and decreased significantly from 1.09 at baseline to a low of 1.04 at 428 days (p<0.0001). Furthermore, change in EAT following bariatric surgery correlated with change in LVei (r=0.43,p=0.0007).

**Conclusions:**

Cardiac volumes show a biphasic response to weight loss, initially becoming smaller and then returning to pre-operative sizes by 1030 days. We propose this is due to an initial reversal of eccentric remodelling followed by reversal of concentric remodelling. Furthermore, we provide evidence for a role of EAT contributing to pericardial restraint, with EAT loss improving markers of pericardial restraint.

## Introduction

Although obesity implies a global increase in total body fat, on an individual basis fat is deposited differentially into specific subcutaneous and visceral compartments of the body and also ectopically within organs (1). It is now becoming clear that the increased cardiovascular risk associated with obesity (2) is not solely the result of increased adiposity, but is also affected by the distribution of this excess fat, with evidence that visceral depots are more strongly linked to increased cardio-metabolic risk than subcutaneous depots (3–5). As a result, the pattern of fat distribution within an individual is likely to provide a better picture of cardiovascular risk than global measures of obesity (6, 7). Furthermore, there is emerging evidence that the greater adverse effect of visceral fat extends beyond cardiovascular risk factors and results in an increased likelihood of left ventricular (LV) hypertrophy (8, 9).

The combination of increased total adipose tissue and skeletal muscle volumes results in higher cardiac output and total blood volume. This leads to LV cavity dilatation and eccentric hypertrophic remodelling (10, 11) secondary to increased wall stress imposed by this cavity dilatation (12, 13). Although this accounts for the eccentric hypertrophic pattern, it is now becoming clear that concentric remodelling also occurs in obesity (14–16). The cause of this concentric remodelling has been shown to be related to insulin resistance (17), hyperleptinaemia (18) and myocardial steatosis (19) driven by visceral adipose tissue (20). In line with this, in diabetes, concentric remodelling with small LV cavity size is commonly reported (17).

Following bariatric surgery, many studies have reported reverse remodelling. LV mass has been universally shown to be reduced following weight loss and proportionally related to weight loss over time (21–24). With the reduction in both subcutaneous (driving volume load and eccentric hypertrophy) and visceral adiposity (driving insulin resistance and concentric remodelling) following bariatric surgery, this would be expected. The results for left ventricular cavity size changes are far less consistent, with some reporting a reduction in LV cavity size (22, 25) whilst others no change (26, 27), or an increase (28). In this case, the early reduction in volume load that accompanies total fat mass reduction would be expected to reduce LV cavity size, however, the reversal of insulin resistance that accompanies bariatric surgery (29), may be expected over time to reduce concentric remodelling and thus increase LV cavity size. Whether a reduction in epicardial adipose tissue allows additional expansion due to relief of external compression (30) or had a local paracrine effect on ventricular muscle given it’s anatomical location (31), is unknown.

We hypothesised that LV geometric remodelling would follow a biphasic time-course characterised initially by LV cavity reduction and reduced LV mass (as eccentric remodelling reverses), followed by relative LV cavity dilation with the return of insulin sensitivity (and reversal of concentric remodelling). We also hypothesised that a reduction in epicardial adipose tissue (EAT) following bariatric surgery would increase space within the pericardium, thus decreasing pericardial restraint. In order to assess these hypotheses, we performed a serial imaging study involving 62 participants who underwent CMR imaging to assess cardiac geometry alongside epicardial and visceral adipose tissue, before and after bariatric surgery. The study included 36 participants who underwent short-term (median 212 days), 37 medium-term (median 428 days) and 32 long-term (median 1030 days) imaging follow up.

## Materials and methods

### Cohort

62 participants (28 gastric bypass, 26 sleeve gastrectomy, 8 adjustable gastric band) were recruited from the bariatric surgery clinics at the Oxford University Hospitals Trust and Metabolic Diseases and Nutrition, Centre Hospitalier Universitaire Nord, Marseille, France and underwent a CMR scan prior to surgery as described below. Participants attended follow up CMR scans at varying timepoints after bariatric surgery, including at a median follow up of 212 days (n=36), 428 days (n=37) and 1030 days (n=32) (43 participants were imaged at two follow up points and 19 participants were imaged at one follow up point). The surgical procedure proportion was similar across timepoints.

The study was approved by the local research ethics committee (NHSREC Ref 15/SC/004, or local REC in Marseille (NCT01284816)), and informed written consent obtained from all volunteers Exclusion criteria included uncontrolled atrial fibrillation, established cardiac disease such as history or symptoms of flow-limiting coronary artery disease, infarction on CMR, severe valvular heart disease, recent change in medications, previous bariatric surgery, and standard contraindications to MR scanning (pregnancy, breastfeeding, implanted metallic devices, severe claustrophobia) All study visits were performed with the volunteer fasted for 8 hours.

### Anthropomorphic and biochemical assessment

Height, weight and body composition were measured using digital scales (InBody 770, InBody Co Ltd, South Korea). Fasting venous blood was taken and biomarkers were analysed by the Oxford University Hospitals clinical biochemistry laboratory according to standardised protocols. Fasting insulin resistance was represented by HOMA-IR ((glucose x insulin)/22.5).

### Magnetic resonance imaging

#### Visceral fat depots

Visceral adipose tissue (VAT) was measured with a 5mm transverse slice at the level of the 5^th^ lumbar vertebral body, using a water-suppressed turbo spin echo (TSE) sequence and analysed with manual contouring as previously described (23).

**Figure 1:**
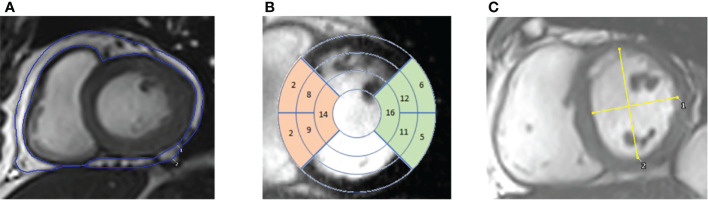
CMR images. **(A)** – contouring of epicardial adipose tissue in the short axis view. **(B)** – 17 segment view overlayed on short axis images. Highlighting the septal segments (in orange) and the lateral segments (in green). **(C)** – Short axis view with measurements of the maximal anterior-posterior (AP) diameter parallel to the septum and maximal septal-lateral (SL) diameter orthogonal to the AP diameter, with left ventricular eccentricity index being calculated by diving AP/SL diameters.

Epicardial adipose tissue (EAT) was manually delineated on the same short axis stack images from the atrioventricular valve annuli to the apex in end-ventricular systole (**Figure 1A**). Surfaces were then summed and multiplied by slice thickness to obtain epicardial adipose tissue volume.

#### Cardiac imaging

Cardiac imaging to quantify ventricular volumes and function was acquired using an SSFP sequence (echo time 1.5ms, repetition time 3ms), which was performed with cardiac triggering and during end-expiratory breath-hold, on a 3T MR system (Tim Trio, Siemens, Munich, Germany/Verio, Siemens, Erlangen, Germany) as previously described (32). Typical SSFP sequence parameters were slice thickness 8 mm, gap 2 mm, retrospective gating, TE 1.5 ms, TR 46 ms, flip-angle 50°, FOV 400 mm, matrix size 256 in frequency encode direction. Endocardial and epicardial left ventricular contours were drawn and analysed using a semi-automated system to produce left ventricular mass (LVM), left ventricular end-diastolic volume (LVEDV) and left ventricular stroke volume (LVSV) (cmr42, Circle Cardiovascular Imaging Inc, Calgary, Canada) as previously described (33). Left atrial volumes (LAV) were acquired from contouring long axis cine images in ventricular end-systole. Average septal and lateral wall thickness was calculated by averaging wall thickness values from AHA segments 2, 3, 8, 9 and 14 (septal) and 5, 6, 11, 12 and 16 (lateral) in ventricular end-diastole (**Figure 1B**). In the short axis view at the level of the papillary muscles in ventricular end-diastole, maximal anterior-posterior (AP) LV diameter parallel to the septum and maximal septal-lateral (SL) distance perpendicular to the septum were measured (**Figure 1C**). Left ventricular eccentricity index (LVei) was calculated by dividing AP length by SL length.

### Statistical analysis

All statistical analysis was performed using GraphPad Prism (GraphPad Software, San Diego, California USA). Data are expressed as means ± standard deviation (SD) unless otherwise stated. For imaging parameters, percentage change relative to baseline was calculated to account for individual variation in parameters. All continuous variables were normally distributed. As not all participants were scanned at each time point, differences between timepoints were assessed using a mixed-effects model with the Geisser-Greenhouse correction. Bivariate correlations were performed to compute Pearson correlation coefficients. When assessing change in parameters (EAT, VAT and LVei) over the course of the study for the correlation analysis, the difference in maximal timepoints was used (e.g. if a participant was scanned at 3 timepoints, the difference in values between the first and final timepoints were used). A probability value of p < 0.05 was considered significant and two-tailed p values were used for all statistics.

## Results

### Baseline characteristics

Patients included in this cohort had a mean age of 44 (SD 9.9) years, with 74% of the participants being female. Average weight was 124.0kg (SD 19.1), average excess body weight was 54.5kg (SD 15.3) and average BMI was 44.6kg/m^2^ (SD 5.3). Participants had an average systolic and diastolic blood pressure of 129.6 mmHg (SD 18.6) and 74.3 mmHg (SD 10.1), respectively. Seven patients (11%) had diagnosed hypertension with four patients receiving medication for hypertension. Fasting venous blood samples revealed an average fasting glucose of 5.9 mmol/L (SD 2.0), total cholesterol of 4.9 mmol/L (SD 1.0), triglycerides of 1.3 mmol/L (SD 0.8) and a HOMA-IR of 4.4 (SD 3.5). Eight (13%) participants had dyslipidaemia and 9 (15%) had diabetes (**Table 1**), with 6 and 5 receiving treatment for these conditions respectively.

**Table 1:** Baseline characteristics of patients undergoing bariatric surgery.

Participants included in analysis (n=62)	
**Age (years)**	44.4 (9.9)
**Sex (females; n (%))**	46 (74%)
**Weight (kg)**	124.0 (19.1)
**BMI (kg/m^2^)**	44.6 (5.3)
**Excess Body Weight (kg)**	54.5 (15.3)
**Systolic Blood Pressure (mmHg)**	129.6 (18.6)
**Diastolic Blood Pressure (mmHg)**	74.3 (10.1)
**Hypertension (n (%))**	7 (11%)
**Fasting Glucose (mmol/L)**	5.9 (2.0)
**Total Cholesterol (mmol/L)**	4.9 (1.0)
**Fasting Triglycerides (mmol/L)**	1.3 (0.8)
**Dyslipidaemia (n (%))**	8 (13%)
**HOMA-IR**	4.4 (3.5)
**Diabetes (n (%))**	9 (15%)

Values are mean (standard deviation) unless otherwise stated. BMI, body mass index, HOMA-IR, homeostatic model assessment of insulin resistance.

### Sequence of body changes following bariatric surgery

**Figure 2:**
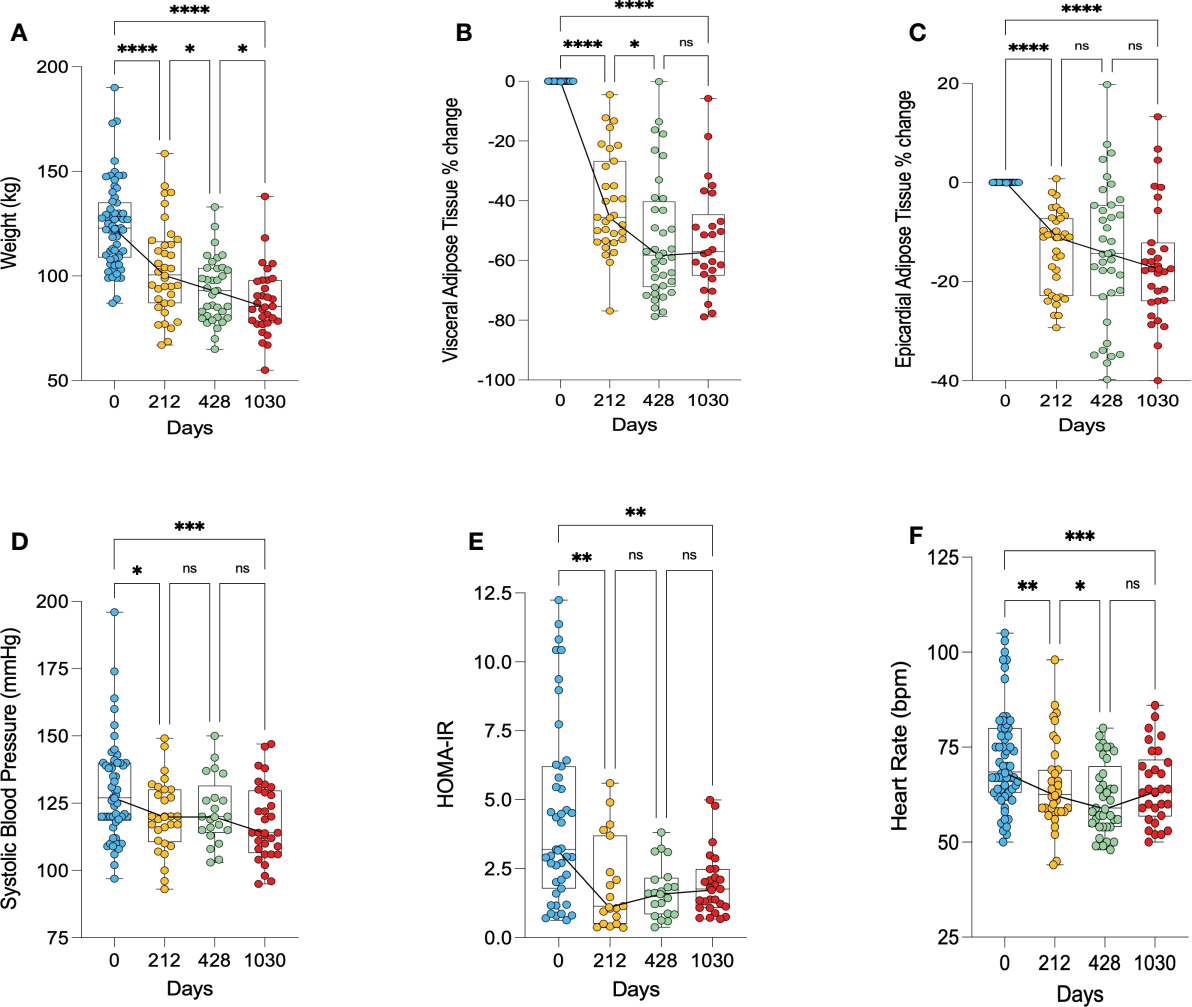
Anthropomorphic changes following bariatric surgery. **(A)** – change in weight, **(B)** – percentage change in visceral adipose tissue, **(C)** – percentage change in epicardial adipose tissue, **(D)** – change in systolic blood pressure, **(E)** – change in HOMA-IR as a marker of insulin resistance, **(F)** – change in heart rate. HOMA-IR, homeostatic model assessment of insulin resistance. * < 0.05, ** < 0.01, ***<0.001, ****<0.0001, ns, Non significant.

Following surgery, participants weight on average decreased from 124kg to 103kg at 212 days (38.9% excess weight loss), before decreasing further to 93kg at 428 days (56.5% excess weight loss) and 88kg at 1030 days (64.7% excess weight loss) (**Figure 2A**).

The majority of VAT was lost in the first 212 days, with an average 43% decrease in VAT (p<0.0001). At the long-term follow up point of 1030 days, there was a 53% decrease in VAT relative to baseline (p<0.0001) (**Figure 2B**). Similarly, the majority of EAT loss occurred in the first 212 days, showing on average a 14% decrease from baseline (p<0.0001). At 1030 days, there was on average a 16% decrease in EAT from baseline (p<0.0001) (**Figure 2C**).

There was a decrease in systolic blood pressure from 130 mmHg pre-operatively to 120 mmHg at 212 days post-surgery (p=0.046) and 118 mmHg at 1030 days (p=0.0005) (**Figure 2D**). Insulin resistance, as measured by HOMA-IR, significantly decreased in the first 212 days (p=0.002), and remained lower at 1030 days (p=0.007) (**Figure 2E**). Heart rate significantly decreased following bariatric surgery, remaining significantly lower at 1030 relative to baseline (p=0.0006) (**Figure 2F**). Three of the 7 participants with hypertension, 2 of the 8 participants with dyslipidaemia, and 7 of the 9 patients with diabetes had resolution of their conditions.

### Cardiac geometry changes following bariatric surgery

**Figure 3:**
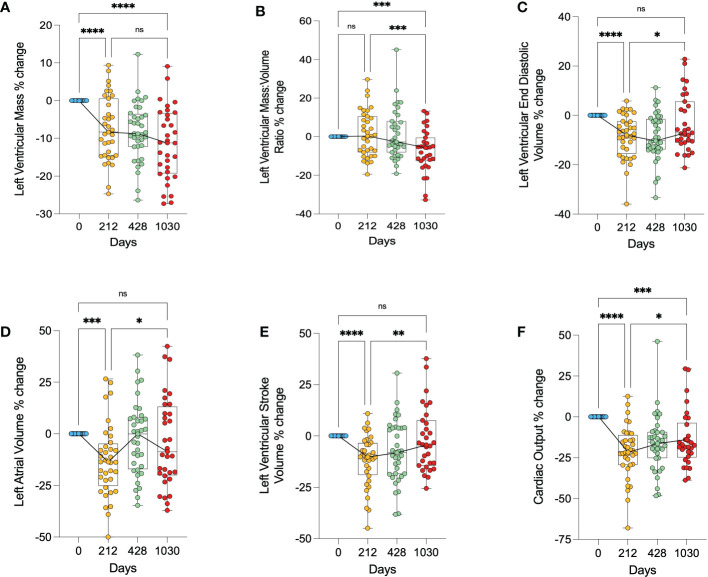
Changes in cardiac geometry following bariatric surgery. **(A)** – percentage change in left ventricular mass, **(B)** – percentage change in left ventricular mass:volume ratio, **(C)** – percentage change in left ventricular end diastolic volume, **(D)** – percentage change in left atrial volume, **(E)** – percentage change in left ventricular stroke volume, **(F)** – percentage change in cardiac output. * < 0.05, ** < 0.01, ***<0.001, ****<0.0001, ns, Non significant.

There was on average a 7.4% decrease in left ventricular mass (LVM) in the first 212 days (p<0.0001), with a further decrease of 12% relative to baseline at 1030 days (p<0.0001) (**Figure 3A**). Left ventricular mass:volume ratio (LVMVR - a marker of concentric remodelling) appeared to only decrease after 428 days, showing on average a 7.3% decrease at day 1030 relative to baseline (p=0.0010) (**Figure 3B**). LVEDV decreased significantly in the short term (day 212) by 8.6% (p<0.0001) but had increased to a volume not significantly different from pre-operative LVEDV by day 1030 (**Figure 3C**). LAV and LVSV also appeared to show this biphasic relationship. Both decreased significantly in the first 212 days (LAV by 13% (p<0.0001) and LVSV by 12% (p<0.0001)) but returned to values not significantly different from baseline at day 1030 (**Figures 3D, E**). Cardiac output decreased on average by 21% during the first 212 days (p<0.0001) and remained significantly lower at 1030 days (p=0.0001), although did show a small increase from 212 days to 1030 days (7.6% points, p=0.0230) (**Figure 3F**). The initial fall in cardiac output correlated with the decrease in both LVEDV (r=0.35, p=0.03) and LAV (r=0.37, p=0.03).

Overall, this shows a biphasic response of the left ventricular and left atrial sizes, initially getting smaller, and then larger, returning to baseline values. It also suggests that whilst LV mass was reduced continually through follow up, initially this loss was proportional to the LV cavity size reduction, and at later timepoints showed a reduction in concentric remodelling.

### Local effect of epicardial adipose tissue on cardiac geometry

**Figure 4:**
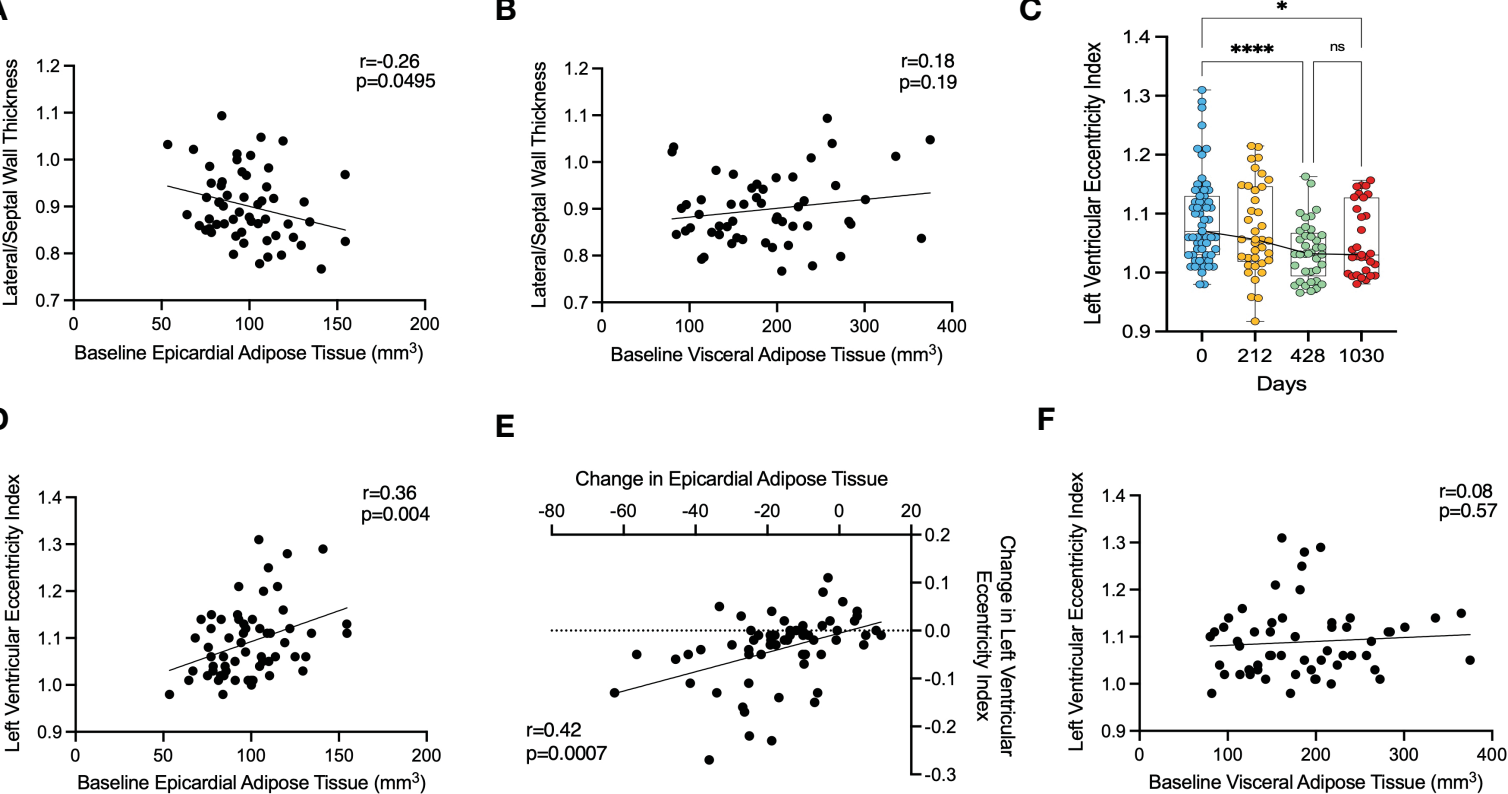
The effect of epicardial adipose tissue on cardiac geometry following bariatric surgery. **(A)** – correlation between baseline lateral/septal wall thickness ratio and baseline epicardial adipose tissue, **(B)** – correlation between baseline lateral/septal wall thickness ratio and baseline visceral adipose tissue, **(C)** – change in left ventricular eccentricity index, **(D)** - correlation between left ventricular eccentricity index and baseline epicardial adipose tissue, **(E)** - correlation between change in left ventricular eccentricity index and change in epicardial adipose tissue, **(F)** – correlation between left ventricular eccentricity index and baseline visceral adipose tissue. *<0.05, *** < 0.001, **** < 0.0001 ns, Non significant.

In order to assess whether epicardial adipose tissue has a local effect on ventricular wall thickness, we examined the relationship between EAT and regional LV thickness. For this we compared the relationship between EAT volume, and the lateral-to-septal wall thickness ratio, since the lateral wall is in close proximity to EAT and the septal wall is not, enabling assessment for regional effects. EAT was weakly, but inversely correlated with a lateral-to-septal wall thickness ratio (r=-0.26, p=0.0495) (**Figure 4A**), suggesting that increased EAT was not related to local LV hypertrophy. However, when looking at the correlation between EAT and lateral or septal wall thickness individually, no relationship was found (r=0.05, p=0.72 and r=0.22, p=0.10, respectively). Baseline VAT was not associated with lateral/septal wall thickness ratio (r=0.18, p=0.19) (**Figure 4B**). Increasing VAT was associated with both increased lateral (r=0.47, p=0.0003) and septal (r=0.34, p=0.0118) wall thickness.

Overall, this would suggest that there was no local effect of EAT on lateral wall thickness, and that the observed relationship was driven more by VAT volume.

### Effects of epicardial adipose tissue on pericardial restraint

Finally, we investigated the left ventricular eccentricity index (LVei) in end-diastole as a marker of ventricular interdependence and thus pericardial restraint. LVei decreased significantly from a mean of 1.09 (SD 0.08) at baseline to 1.04 (SD 0.05) at 428 days (p<0.0001) and was maintained with no further reduction at 1030 days (mean 1.06, SD 0.06, p=0.048) (**Figure 4C**). LVei appeared to be positively correlated with baseline EAT (r=0.37, p=0.0040) (**Figure 4D**) and moreover, change in EAT over maximal timepoints was correlated with change in LVei over maximal timepoints (r=0.43, p=0.0007) (**Figure 4E**). On the other hand, baseline VAT did not correlate with LVei (r=0.08, p=0.5713) (**Figure 4F**) and change in VAT over maximal timepoints was not correlated with change in LVei over maximal timepoints (r=0.10, p=0.4627).

This would suggest that epicardial adipose tissue causes some pericardial mass effect and pericardial restraint, which is improved with weight loss.

## Discussion

**Figure 5:**
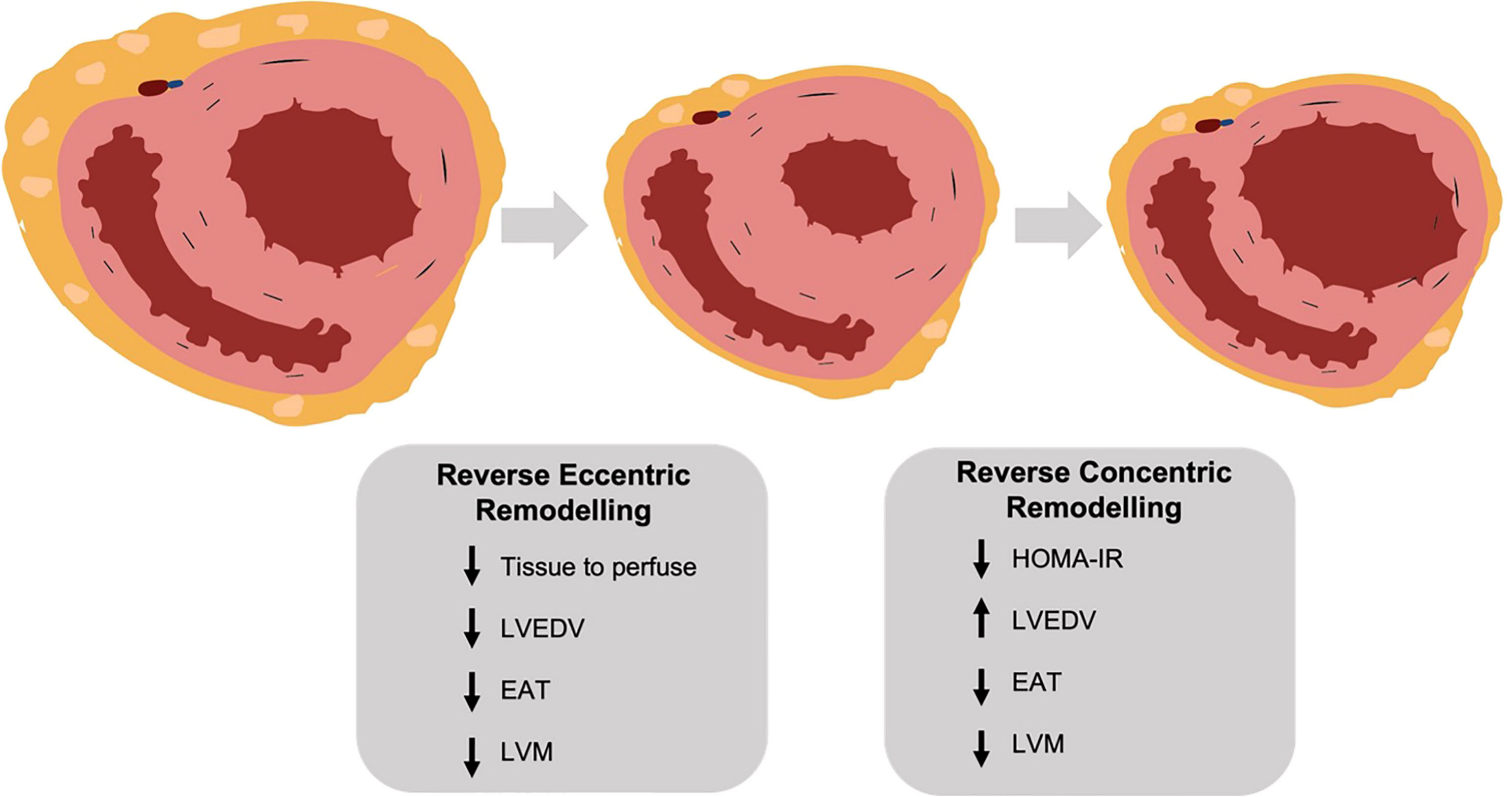
(central figure) – following bariatric surgery the left ventricle undergoes a biphasic remodelling response. Initially there is a reversal of eccentric remodelling as total body tissue to perfuse falls, with reductions in left ventricular mass, end diastolic volume and epicardial adipose tissue. In the longer term there is a reversal of concentric remodelling as insulin resistance (HOMA-IR) decreases, with a further fall in left ventricular mass but an increase in end diastolic volume. LVEDV, left ventricular end diastolic volume; EAT, epicardial adipose tissue; LVM, left ventricular mass; HOMA-IR, homeostatic model assessment of insulin resistance. Created with BioRender.com.

The cardiac remodelling in obesity is characterised by mixed eccentric and left ventricular hypertrophy and left atrial dilatation. In this study we show that following bariatric surgery there is an initial reduction in LV and LA cavity size mirroring the reduction in cardiac output, followed by an increase in chamber size with longer-term assessment. Associated with this is a reduction in epicardial adipose tissue and reduced LV eccentricity index, indicating reduced pericardial constraint. Additionally, we show that whilst LV mass was reduced continually throughout follow up, in early follow up this loss was proportional to the LV cavity size reduction, and at later timepoints a reduction in concentric remodelling is seen. Furthermore, we show that epicardial adipose tissue has no local effect on LV hypertrophy in obesity. A such we provide evidence for a biphasic cardiac response to weight loss (summarised in **Figure 5**).

### Changes in left ventricular geometry following weight loss

#### Eccentric LV remodelling

As expected, in our study significant decreases in total body weight and VAT were seen quickly following bariatric surgery. This rapid reduction in total body tissue to perfuse decreases the cardiac output requirement which was also observed. We see here that this reduction in cardiac output is a result of both decreased heart rate and LV end-diastolic volume, leading to reduced stroke volume. With a smaller stroke volume and LV cavity size it would be expected that LV mass would also fall, and we observe this to be the case in this study with the fall in LV mass being proportional to the reduction in LV cavity size. This initial reversal of eccentric remodelling occurs early post bariatric surgery with the majority of the change seen at the earliest timepoint of 212 days. These results agree with a prior study relating regression in LV mass to reductions in VAT (34), and are particularly important given the increasing recognition for VAT playing a pathophysiology role in obesity-related heart failure (35).

#### Concentric LV remodelling

However, the hormonal milieu in obesity also contributes to LV hypertrophy, with insulin and leptin both stimulating myocyte hypertrophy. Indeed, we have shown that diabetes and insulin resistance is associated with smaller LV cavity size and concentric remodelling (19). When put together with elevation in blood pressure, which is commonly associated with obesity, concentric remodelling is also observed with increased body mass index (14–16). As weight loss is known to reduce leptin, insulin and blood pressure (17, 18, 36), this aspect of LV remodelling should also reverse as shown in a previous study (37). In line with this we observed a significant decrease in insulin resistance (HOMA-IR) and systolic blood pressure and a reduction in LV mass-to-volume ratio at the longest term. The fall in insulin resistance in the short term is likely to take time to translate into reversal of concentric remodelling, explaining the delayed improvement in concentric remodelling. The reductions in LV remodelling, in tandem with decreases in volume overload, may be associated with salutary reductions in cardiac filling pressures, as previously reported following weight loss (38).

#### A biphasic ventricular response

Following bariatric surgery, studies have reported conflicting results regarding LV end-diastolic volume, with increased (28), decreased (22, 25) and no change being reported (26, 27). This study shows a biphasic response of the LV cavity following weight loss, with the initially observed decrease in LV cavity size being followed by a longer-term increase in cavity size, back to the baseline size. We propose that this cavity size increase is due to the improvement in insulin sensitivity, reduction in LV concentric remodelling, and the reduction in epicardial adipose tissue, that allows the LV cavity to expand. In line with this local volume effect of EAT, the significant decrease in EAT observed in this study was correlated with improvements in LV eccentricity index, a marker of ventricular interdependence and pericardial restraint (30, 39, 40). As such, the heterogeneity of reported changes in LV cavity size following bariatric surgery may simply be due to reporting at variable time intervals. In keeping with this, those that have reported up to one year after surgery (22, 25), in general report reduced LV cavity size, and those with no change or increase tend to report over two years from surgery (28, 37, 41).

#### Left atrial geometry and weight loss

Similar heterogeneity exists in reports of LA cavity size following bariatric surgery (42). In this study, LA cavity size followed a similar trajectory to LV cavity size, initially falling in size and then increasing from 212 days onwards. Previous studies have also shown a decrease in LA volume within 1 year of bariatric surgery (43, 44), with other studies reporting no change in LA volume after 1 year (41, 45, 46), and increased LA volume after 5 years (34). Whilst the initial reduction in size follows the reduction in cardiac output, as for the LV, the loss of EAT from the pericardial space may again allow further space for this LA cavity dilation to occur. It may also be that we are simply observing LA enlargement over time as a result of normal ageing (47) which is likely to be greater in a population with obesity (48). Despite observing no change in LA volume over the longer-term post bariatric surgery in this study, we feel it is likely that this still represents an improvement relative to those who don’t undergo weight loss who would be expected to have an increase in LA volume (46).

#### Local effect of epicardial adipose tissue

EAT is a visceral fat depot located entirely within the pericardium and thus is in direct contact with the myocardium. In keeping with its role as an ectopic fat depot, studies have shown that increased EAT is associated with increased myocardial steatosis (49, 50). However, beyond its role as a fat storage depot, EAT is being increasing recognised as a metabolically active tissue, with paracrine effects on the myocardium through adipokine release (51). Given that the lateral wall of the left ventricle is in direct contact with EAT, whilst the septal wall is not, we hypothesised that if a local paracrine hypertrophic effect exists, a difference in wall thickness at these two locations would be observed. Interestingly we found no evidence for such a difference between lateral and septal wall thickness, suggesting that a local hypertrophic effect does not exist.

## Limitations

This study has a number of limitations. Firstly it is a relatively small sample size of 62, with variable follow up time points. Furthermore, participants in this cohort underwent different types of surgery and this may affect the amount of weight lost. Whilst this population was a relatively healthy cohort, some participants did have hypertension (11%) or diabetes (15%), and these conditions themselves or their treatment may have an impact on cardiac geometry. There was also significantly more females (74%) than males in this cohort, and this too may have an impact on cardiac geometry.

## Conclusion

In summary, in this serial imaging study we provide evidence for a biphasic response in LV and LA remodelling following weight loss induced by bariatric surgery. This may be explained by an initial reversal of eccentric remodelling given the decreased demands of the body from global tissue loss, followed by a longer-term reversal of concentric remodelling as insulin sensitivity is restored over a longer duration of time. We also provide evidence for a role of EAT in contributing to pericardial restraint in the setting of obesity, and furthermore show that decreasing EAT correlates with improved markers of pericardial restraint. This study provides insights into how bariatric surgery effects the left ventricle and atrium, and provides an explanation for the inconsistent results previously reported in the field. Furthermore, it adds more evidence to support the beneficial role of decreasing EAT on cardiac geometry and function.

## Data availability statement

The raw data supporting the conclusions of this article will be made available by the authors, without undue reservation.

## Ethics statement

The studies involving human participants were reviewed and approved by the local research ethics committee (NHSREC Ref 15/SC/004, or local REC in Marseille (NCT01284816)). The patients/participants provided their written informed consent to participate in this study.

## Author contributions

JH, IA and OR conceptualised and designed the study. Data was collected by IA, AL, JR, MB, FK, AS, BS, JB, TB. Data analysis was conducted by JH, OD and OR, and BB, AD, BG, SN and OR contributed to data interpretation. The manuscript was written by JH and OR. All authors contributed to the article and approved the submitted version.
